# Reply to: Hypoxia treatment of Parkinson’s disease may disrupt the circadian system

**DOI:** 10.1186/s12883-023-03281-9

**Published:** 2023-06-19

**Authors:** Jules M. Janssen Daalen, Marjan J. Meinders, Isabel R. Straatsma, Philip N. Ainslie, Dick H. J. Thijssen, Bastiaan R. Bloem

**Affiliations:** 1 Center of Expertise for Parkinson & Movement Disorders, Nijmegen, The Netherlands; 2 Department of Neurology, Radboud University Medical Center, Donders Institute for Brain, Cognition and Behavior, Nijmegen, The Netherlands; 3 Department of Physiology, Department of Medical BioSciences, Radboud University Medical Center, Nijmegen, The Netherlands; 4 IQ Healthcare, Radboud Institute for Health Sciences, Radboud University Medical Center, Nijmegen, The Netherlands; 5 Center for Heart, Lung and Vascular Health, School of Health and Exercise Sciences, University of British Columbia, Kelowna, Canada

## Background

### Introduction

In recent years, increasing attention has been given to hypoxia-based treatment for persons with neurodegenerative and mitochondrial disease, as reflected by the significant rise in publications from basic [1], preclinical [2] and clinical [3, 4] research groups. Hypoxia treatment is based on the idea of hypoxic conditioning and adaptations induced by hypoxia. Recently, we published a protocol paper to assess the safety, feasibility, and acute symptomatic effects of single sessions of continuous and intermittent hypoxia (for 45 min, at FiO2 0.133 and 0.163) in persons with Parkinson’s disease (PD) [3].

In Coste & Touitou’s recent correspondence [5] to our protocol [6], they highlighted the potential for circadian rhythm disturbances induced by hypoxia in PD. This interesting insight is based on their two different studies, in which a phase shift in circadian rhythm (as measured by cortisol and melatonin) was observed after eight-hours-long ‘chronic’ exposure to hypoxia [7, 8]. Coste & Touitou [6] carefully considered that hypoxia-based interventions could therefore induce changes in circadian rhythm, and this may in turn affect the outcome of these interventions. Here, we discuss important differences between chronic hypoxia, which resembles hypoxia as a disease model for sleep apnea, and hypoxic conditioning.

### Hypoxia: disease model or disease-modifying potential?

Neurophysiological responses to hypoxia are complex and vary depending on dose, duration and frequency (reviewed in [9]). The first important distinction between chronic hypoxia and hypoxia-based treatment is the difference in experimental design. While chronic hypoxia interventions induce hours-long hypoxia, therapeutic hypoxia-based interventions are based on the principle of hypoxic conditioning. Hypoxic conditioning effects are induced by moderate, relatively brief, and repeated exposure to a hypoxic stimulus. This controlled administration is suggested to lead to the activation of antioxidant pathways through HIF-1-dependent and independent pathways, including the Nrf2-Keap1 signaling pathway. Importantly, activation of these pathways does not appear to cause significant enduring oxidative stress [10, 11]. Indeed, hypoxic conditioning might protect against oxidative stress and subsequent neuro-inflammation from later stressors [12–14]. Nevertheless, it is likely that hypoxic conditioning interventions have a narrow therapeutic window, which has been reviewed comprehensively previously [15]. We know of no published evidence that brief (< 60 min) exposure to IH causes enduring cardiovascular adaptations or sleep disturbances in humans.

Chronic intermittent hypoxia (CIH) is the main experimental model used to investigate the effects of obstructive sleep apnea (OSA) on neurodegeneration [16]. OSA is a disorder characterized by recurrent episodes of partial or complete airway obstruction, resulting in intermittent hypoxia during nocturnal sleep (thus typically imposed for 7–9 h) where oxygen saturations below 80% are common [17]. As suggested by Coste & Touitou [6], an hours-long experimental protocol may adversely affect the circadian rhythm, either by disturbed sleep or due to persistent sympathetic activation [18, 19]. Although a causal relation between sleep apnea and PD has not been established in humans, associations between OSA incidence and PD risk have been reported [20]. Moreover, preclinical evidence is strongly suggestive of sleep apnea inducing nigrostriatal degeneration [21, 22].

The disparity between chronic hypoxia and hypoxic conditioning might stem from differences at the molecular level. Although both chronic hypoxia and hypoxic conditioning activate the HIF-1 pathway, chronic hypoxia induces enduring oxidative stress and sympathetic activation compared to hypoxia conditioning, adversely affecting cardiovascular health [16, 23]. Furthermore, chronic hypoxia induces prolonged NFkB pathway activation, which is a primary driver of neuroinflammation. Indeed, NFkB and downstream pathways such as IL-6 and IL-1β are presumed instigators of sleep apnea-induced neurodegeneration [24].

### Current trial experience

Based on preclinical evidence on the effects of hypoxic conditioning in neurodegenerative diseases [1, 4, 25], we initiated the first hypoxia trial in people with PD [3]. The aim of this currently ongoing double-blinded placebo-controlled study is to assess the safety, feasibility and acute responses of hypoxic interventions in individuals with PD. For the first time, this will allow us to directly investigate the physiological, respiratory and symptom responses to hypoxia in this population. We investigate the acute and delayed response (up to three days post-intervention) to four different hypoxia interventions and a placebo intervention, all with a 45-min duration. Hypoxia interventions are either continuous or intermittent (5 min of hypoxia interspersed with normoxia), at a fraction of oxygen (FiO2) of 0.133 or 0.163. The trial is conducted in 20 individuals with Hoehn & Yahr stage 1.5 to 3 and consists of multiple N-of-1 trials, which allows each participant to be his or her own control and thereby allows for intra-individual outcome analysis.

To address the remarks that were raised in the Comment by Coste & Touitou, we have investigated the amount of reported adverse events (AEs) to date in our study, as well as the nature of AEs per protocol [3]. Across four different hypoxia protocols and one placebo, three sleep-related AEs occurred, two of which were related to restlessness and REM-sleep behaviour disorder. Sleep-related AEs occurred in three different protocols, one of which was a placebo. Therefore, the incidence of sleep-related AEs does not seem to be higher in hypoxia protocols in our study.

In addition to sleep-related AEs, we evaluated sleep quality in our study population as part of non-motor symptom severity scores on a 4-point Likert scale (zero indicating worst sleep quality). The mean sleep quality rating score in the week following a placebo intervention was 3.0 (SD 0.9) and there was no significant difference with hypoxia interventions (mean sleep quality rating score ranged between 2.9 and 3.2). We are not aware of any other studies reporting an association between the timing of hypoxia conditioning and subsequent circadian disturbances. A potential implication of Coste & Touitou’s raised points is that hypoxia administration in the morning might be preferable. As this was also the case in our current study, we cannot draw conclusions on time-dependent circadian disturbances. Taken together, our data do not show an adverse effect of hypoxia interventions on sleep-related events or sleep quality.

Lastly, in our study we will also monitor vital parameters, including arterial blood gas, blood pressure, heart rate (variability), and serum cortisol in the acute phase of administering both intermittent and continuous hypoxia for 45 min. These data will provide insight into the influence of hypoxia on the acute and subacute sympathetic system activation and stress response. However, despite these measures, and in light of Coste & Touitou’s correspondence [6], we think it is appropriate to include an exploratory outcome for sleep quality and circadian rhythm to any follow-up studies in PD hypoxia trials. Further safety and response-related results from this phase 1 study will be addressed in separate reports. Follow-up trials will investigate the effects of hypoxia conditioning, administered multiple times per week.

### Conclusion

Hypoxia conditioning is a potentially novel treatment strategy for mitochondrial and neurodegenerative diseases and differs from hypoxia as a disease model for ischemia and obstructive sleep apnea in a number of key aspects. Importantly, these differences determine the molecular pathways that are induced at a clinically relevant level and, ultimately, the consequences of long-term application. For that reason, it is essential to carefully consider the hypoxic dose, duration and administration frequency when designing clinical trials. Furthermore, monitoring of physiological parameters and induction of downstream target mechanisms is necessary to determine the therapeutic window. Although our preliminary results do not support an adverse effect of hypoxia interventions in persons with PD, further exploration of the effects of hypoxia on circadian rhythm may be warranted in future studies.

## Data Availability

Not applicable.
